# Supplementary material for: Shared associations identify causal relationships between gene expression and immune cell phenotypes
Source: Commun Biol. 2021 Mar 4;4:279. doi: 10.1038/s42003-021-01823-w (PMC7933159; doi:10.1038/s42003-021-01823-w)
Supplement: Supplementary file 2 — Supplementary Information [file 42003_2021_1823_MOESM2_ESM.pdf]

Supplementary material for “Shared associations identify causal relationships between gene expression and immune cell phenotypes”

Christiane Gasperi, Sung Chun, Shamil R. Sunyaev, Chris Cotsapas

Supplementary Figure 1: Number of independent association signals per gene expression trait and locus 1

Supplementary Figure 2: The power to detect shared associations depends on the strength of association to the secondary trait 2

Supplementary Figure 3: Shared genetic association on chromosome 1 for L-selectin (CD62L) in eosinophils and *SELL* expression in neutrophils, monocytes and T cells 3

Supplementary Figure 4: Shared genetic association on chromosome 1 for *CR2* expression in T cells and CD21 in three different B cell subpopulations 4

Supplementary Figure 5: Phenotypic variance of the expression of L-selectin in eosinophils explained by the polygenic risk scores (PRS) of four different genes with a shared genetic effect and by the combination of the PRS of these genes 5

Supplementary Figures 6: *FCRLB* expression in monocytes and CD16 in CD16hi NK cells do not share an association on chromosome 1 6

Supplementary Figures 7: *RPS23P9* expression in neutrophils and HLA-DR in CD8a+CD16hi NK cells do not share an association on chromosome 1 7

Supplementary Figure 8: Shared genetic association on chromosome 3 for *RPS20P15* expression in monocytes and the number of naïve CD4+ T cells 8

Supplementary Figure 9: Shared genetic association on chromosome 13 for *CARS2* expression in neutrophils and the CD4/CD8 ratio 9

Supplementary Figure 10: Shared genetic association on chromosome 12 for *CLEC4C* expression in neutrophils and monocytes and the number of cDC3 10

Supplementary Figure 11: Shared genetic association on chromosome 12 for *ZNF780A* expression in T cells and monocytes and CD69 in HLA-DR+CD16+ NK cells 11

Supplementary Figure 12: Six immune phenotypes were not normally distributed after rank transformation 12

Supplementary Figure 13: Converting two quantitative immune phenotypes to dichotomous traits 13

Supplementary Figure 14: Schematic of the analysis windows used to identify shared genetic effects between immune phenotypes and gene expression traits 14

Supplementary Table 1: Flow cytometry measurements for 166 immune phenotypes provided by the Milieu Intérieur project 15

Supplementary Table 2: Polygenic risk score (PRS) results without conditioning on shared genetic effects 21

Supplementary Figure 1: Number of independent association signals per gene expression trait and locus

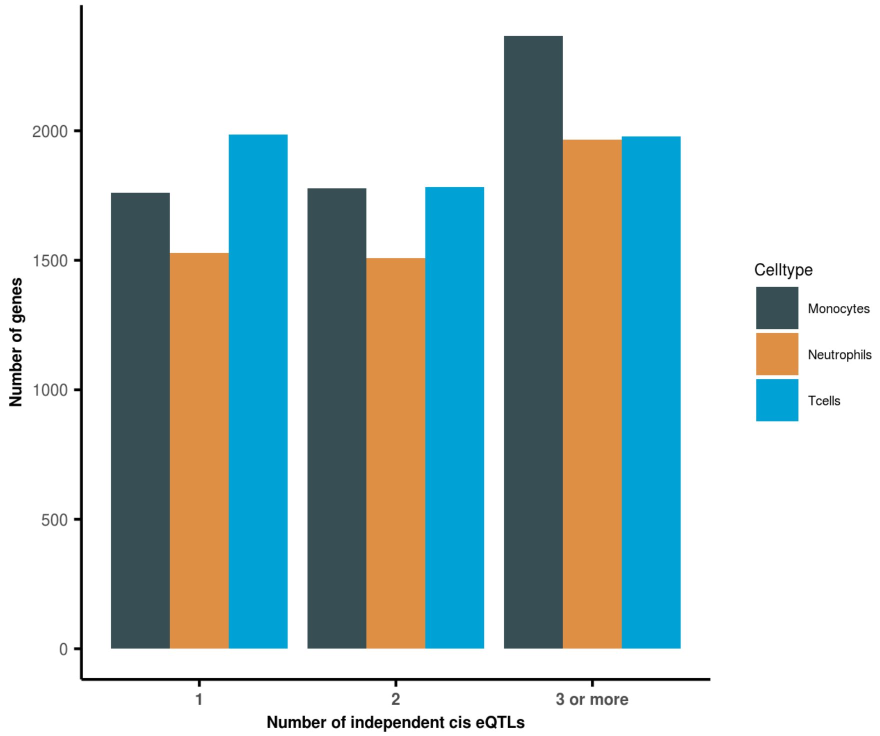

We performed *cis*-expression quantiative trait locus (c*is*-eQTL) analysis on 7,365 genes with a transcription start site (TSS) within 1 megabase from an immune phenotype lead SNP. For 5,906, 5,004 and 5,748 genes we found at least one *cis*-eQTL (within 1.2 megabases from the TSS) in monocytes (gray), neutrophils (yellow) and T cells (blue), respectively. For about two-thirds of these genes we could identify more than one independent *cis*-eQTL.

Supplementary Figure 2: The power to detect shared associations depends on the strength of association to the secondary trait

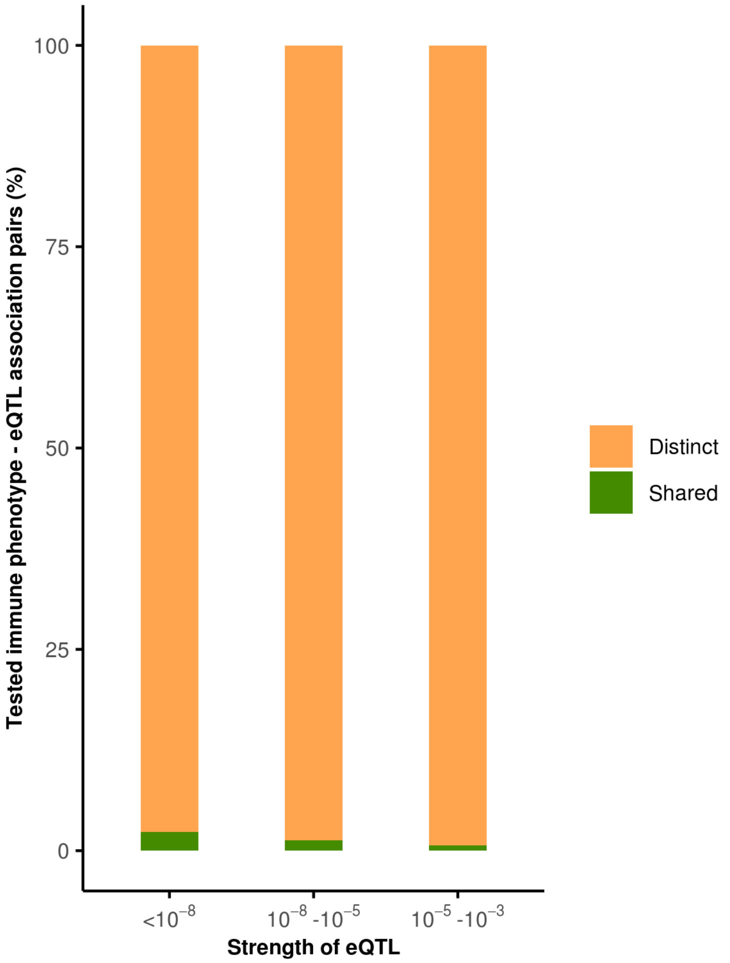

We find evidence that in most cases of colocalized expression quantitative trait loci (eQTLs) and immune phenotypes associations the genetic effects underlying these associations are distinct (orange). The proportion of shared genetic effects (green) we are able to detect depends on the strength of the eQTL association and is highest for strong eQTLs with association $p < 10^{-8}$.

Supplementary Figure 3: Shared genetic association on chromosome 1 for L-selectin (CD62L) in eosinophils and *SELL* expression in neutrophils, monocytes and T cells

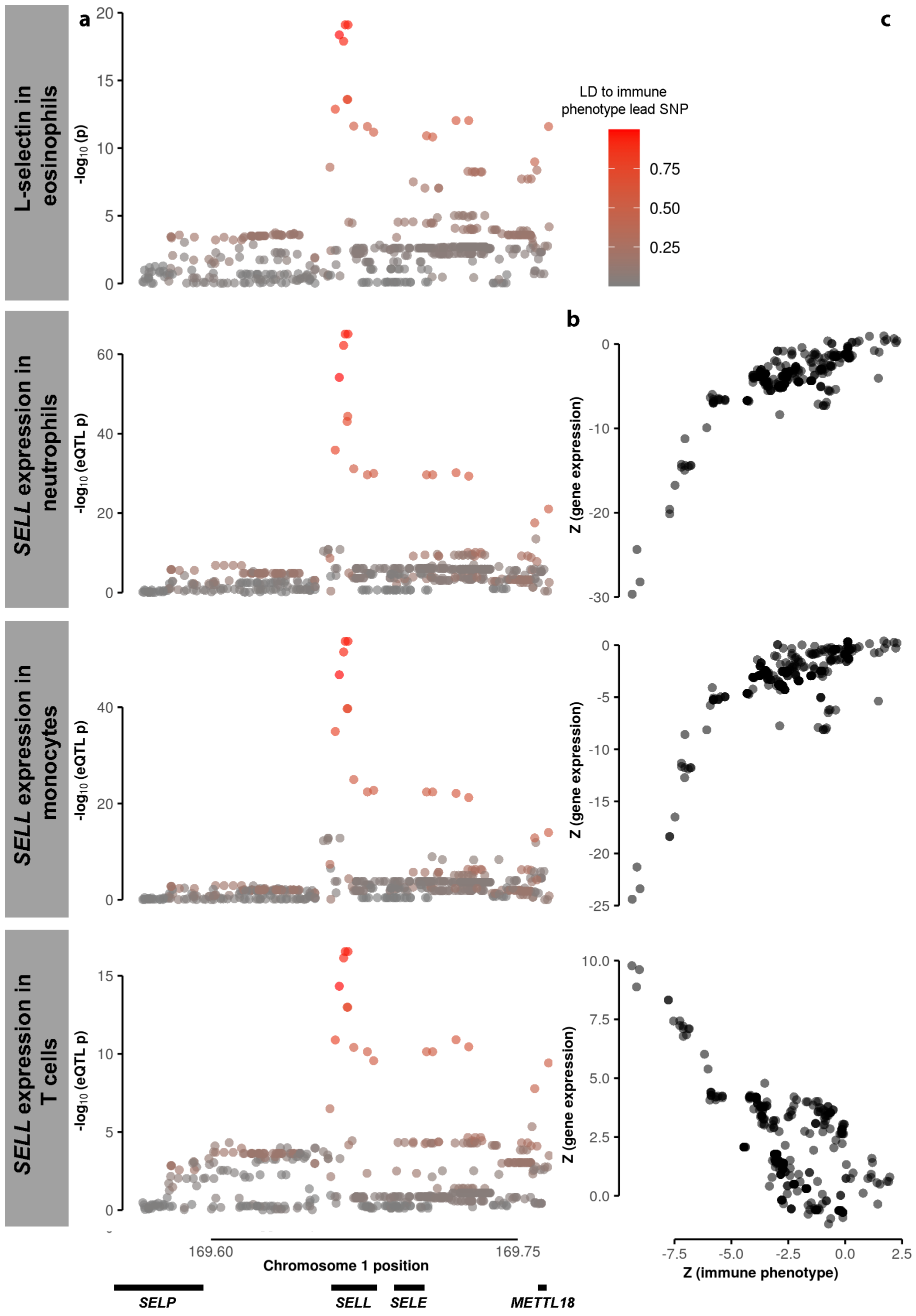

We find a shared genetic association with mean fluorescence intensity (MFI) of L-selectin (CD62L) in neutrophils and with *SELL* expression in neutrophils, monocytes and T cells (a, shaded by linkage disequilibrium (LD, r^2) to the immune phenotype lead single nucleotide polymorphism (SNP)). The association Z statistics of the MFI of L-selectin in neutrophils and *SELL* expression in the three different cell types are strongly correlated (b). (c) shows the MFI of L-selectin and *SELL* expression in the three different cell types (both rank transformed) per genotype of the lead variant rs4987369.

Supplementary Figure 4: Shared genetic association on chromosome 1 for *CR2* expression in T cells and CD21 in three different B cell subpopulations

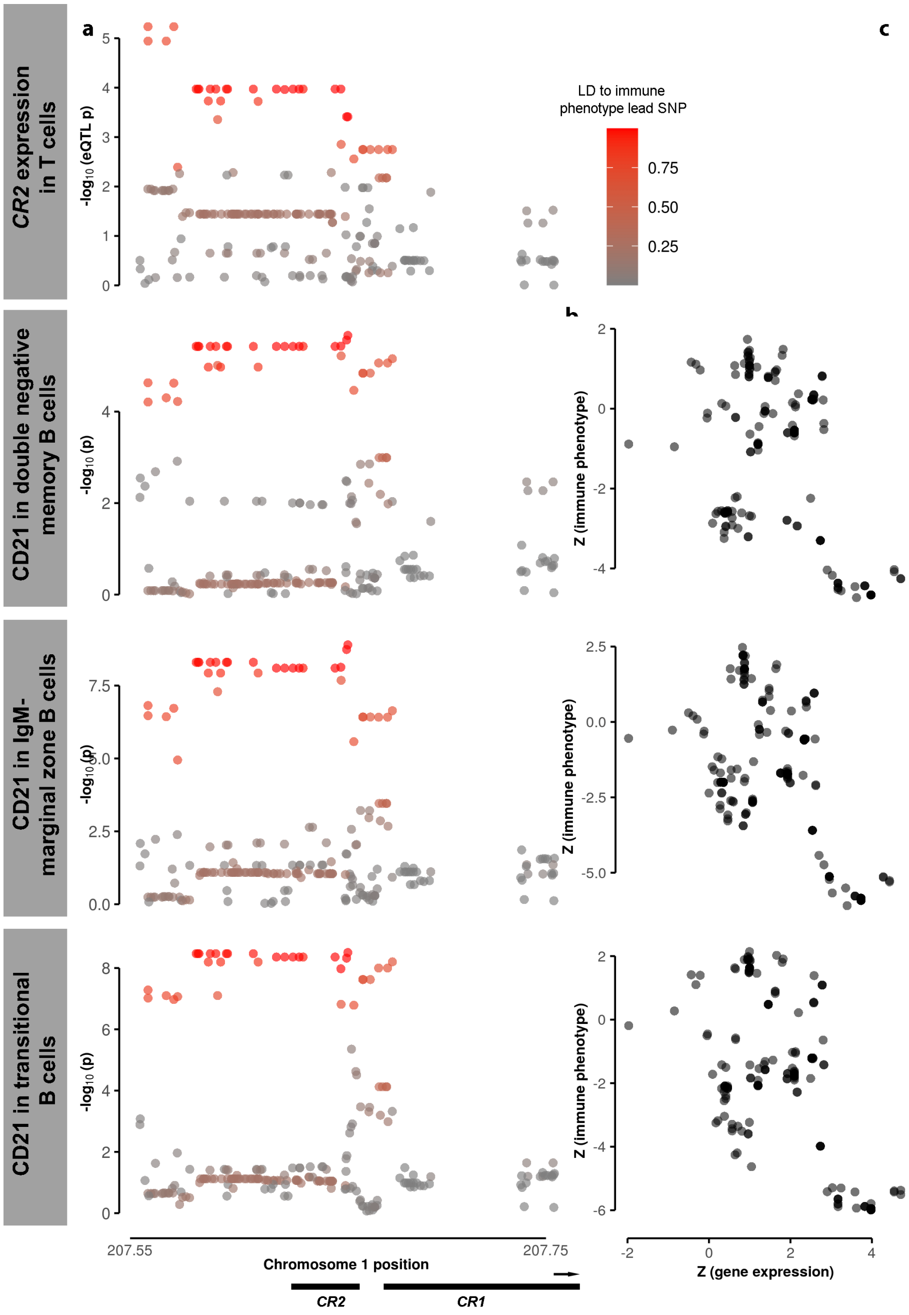

The association signal of the mean fluorescence intensity (MFI) of CD21 in three different B cell subpopulations - double negative memory B cells, IgM- marginal zone B cells and transitional B cells - at a genetic locus on chromosome 1 is consistent with the association signal to *CR2* expression in T cells (a, shaded by linkage disequilibrium (LD, r^2) to the immune phenotype lead single nucleotide polymorphism (SNP)). The association Z statistics of the MFI of CD21 in the three B cell subpopulations and *CR2* expression in T cells are strongly correlated (b). (c) shows the MFI of CD21 in the three B cell subpopulation and the level of *CR2* expression in T cells (all rank transformed) per genotype of the lead variant rs12059417.

Supplementary Figure 5: Phenotypic variance of the expression of L-selectin in eosinophils explained by the polygenic risk scores (PRS) of four different genes with a shared genetic effect and by the combination of the PRS of these genes

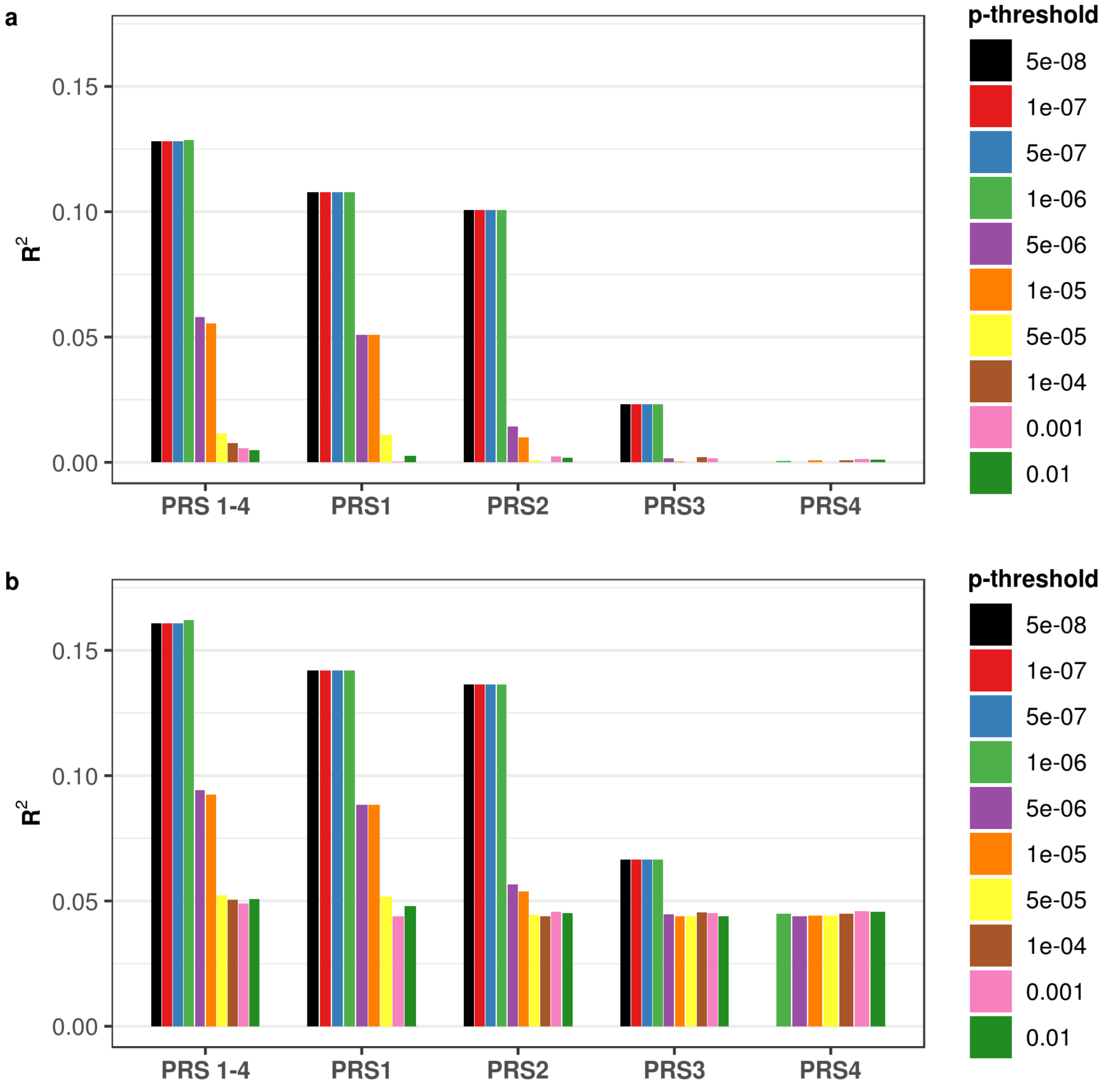

The phenotypic variance (R^2) of the expression of L-selectin (CD62L) in eosinophils explained by single polygenic risk scores (PRS) for four different genes with evidence for a shared genetic effect with the immune phenotype - *SELL* (PRS1), *SELP* (PRS2), *TNF786* (PRS3) and the pseudogene *EEF1A1P2* (PRS4) as well as by the combination of all four PRS (PRS 1-4) has been calculated by linear regression. Regression models were run without any other predictors (A) or including age, sex, CMV status and smoking status as well as the first five principal components in the regresion models (B). The phenotypic variance explained was highest when including the PRS scores of all four genes with evidence for a shared genetic effect with the expression of L-selectin (CD62L) in eosinophils into the regression models. The selection of single nucleotide polymorphisms (SNPs) included in the PRS calculation was based on ten eQTL p-thresholds (see color legend).

Supplementary Figures 6: *FCRLB* expression in monocytes and CD16 in CD16hi NK cells do not share an association on chromosome 1

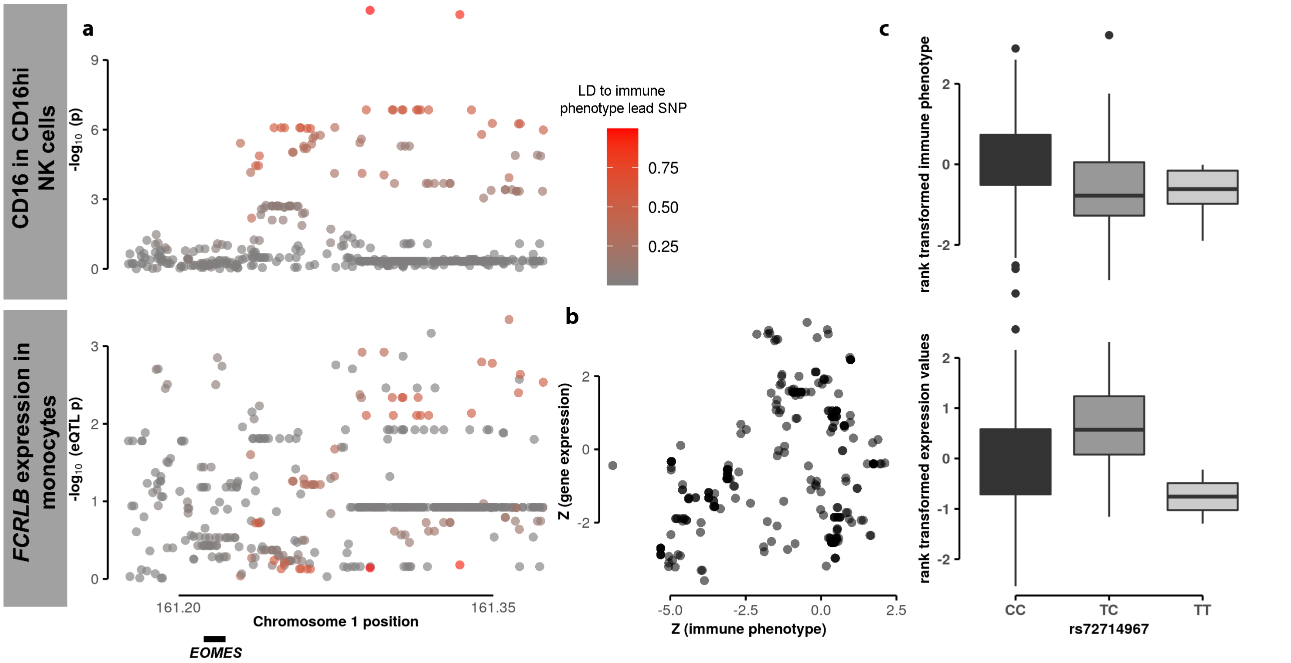

A locus on chromosome 1 without a shared effect between two traits (*FCRLB* expression in monocytes and CD16 in CD16hi NK cells), where gene expression polygenic risk scores (PRS) explain an appreciable fraction of immune trait variance (R^2_{PRS} = 0.06 for a p-value threshold of 5×10^{-8} for the PRS calculation). (a) shows the association signals for both traits (shaded by linkage disequilibrium (LD, r^2) to the immune phenotype lead single nucleotide polymorphism (SNP). (b) shows the association Z statistics for both traits, and (c) shows the immune phenotype (CD16 in CD16hi NK cells) and the gene expression levels (*FCRLB* expression in monocytes) per genotype of the lead variant rs72714967.

Supplementary Figures 7: *RPS23P9* expression in neutrophils and HLA-DR in CD8a+CD16hi NK cells do not share an association on chromosome 1

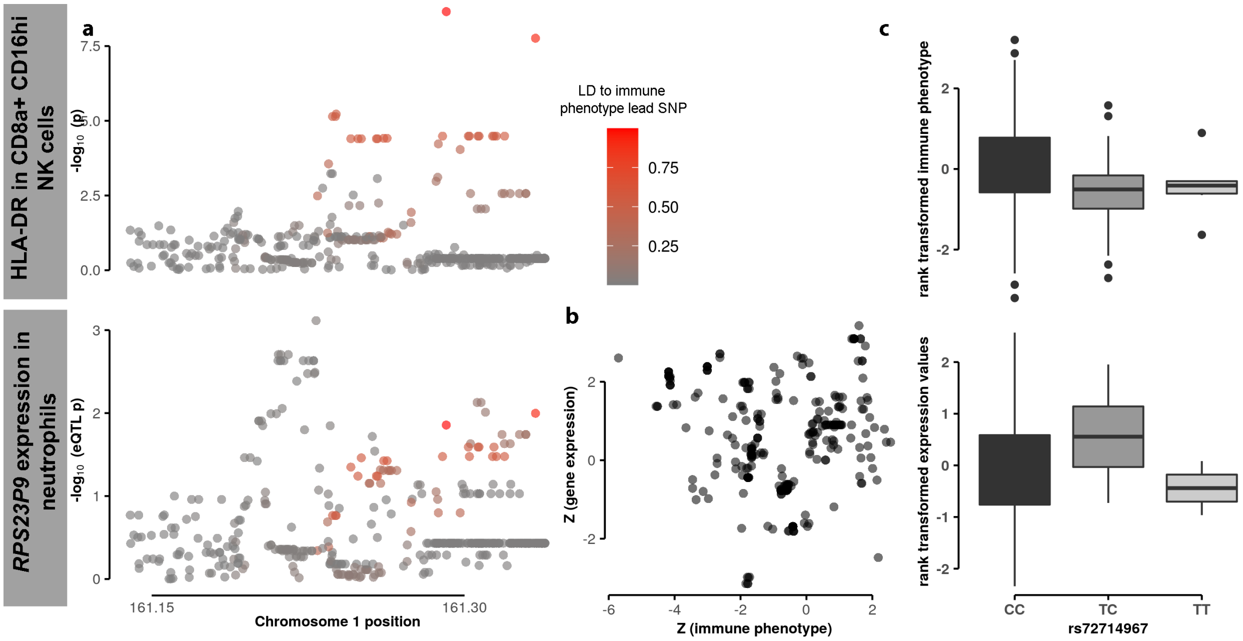

A locus on chromosome 1 without a shared effect between two traits (*RPS23P9* expression in neutrophils and HLA-DR in CD8a+CD16hi NK cells), where gene expression polygenic risk scores (PRS) explain an appreciable fraction of immune trait variance (R^2_{PRS} = 0.05 for a p-value threshold of 5×10^{-8} for the PRS calculation). (a) shows the association signals for both traits (shaded by linkage disequilibrium (LD, r^2) to the immune phenotype lead single nucleotide polymorphism (SNP). (b) shows the association Z statistics for both traits, and (c) shows the immune phenotype (HLA-DR in CD8a+CD16hi NK cells) and the gene expression levels (*RPS23P9* expression in neutrophils) per genotype of the lead variant rs72714967.

Supplementary Figure 8: Shared genetic association on chromosome 3 for *RPS20P15* expression in monocytes and the number of naïve CD4+ T cells

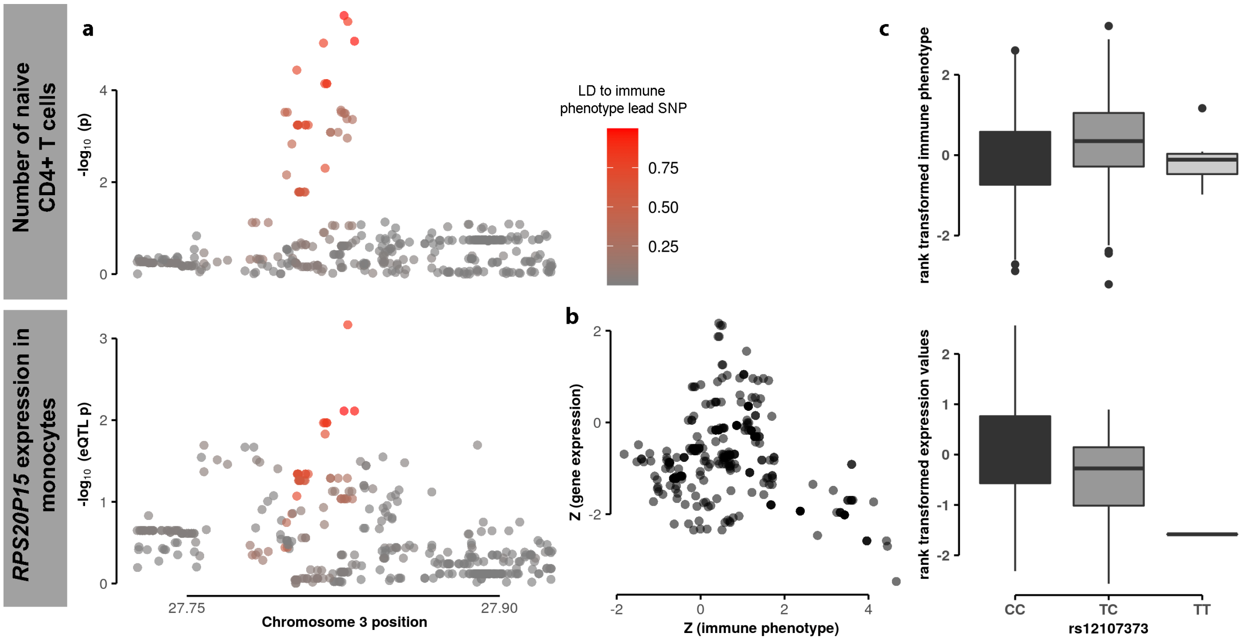

The association signal of the number of naïve CD4+ T cells at a genetic locus on chromosome 3 is consistent with the association signal to *RPS20P15* expression in monocytes (a, shaded by linkage disequilibrium (LD, r^2) to the immune phenotype lead single nucleotide polymorphism (SNP)). The association Z statistics of the number of naïve CD4+ T cells and *RPS20P15* expression in monocytes are strongly correlated (b). (c) shows the number of naïve CD4+ T cells and the level of *RPS20P15* expression in monocytes per genotype of the lead variant rs12107373.

Supplementary Figure 9: Shared genetic association on chromosome 13 for *CARS2* expression in neutrophils and the CD4/CD8 ratio

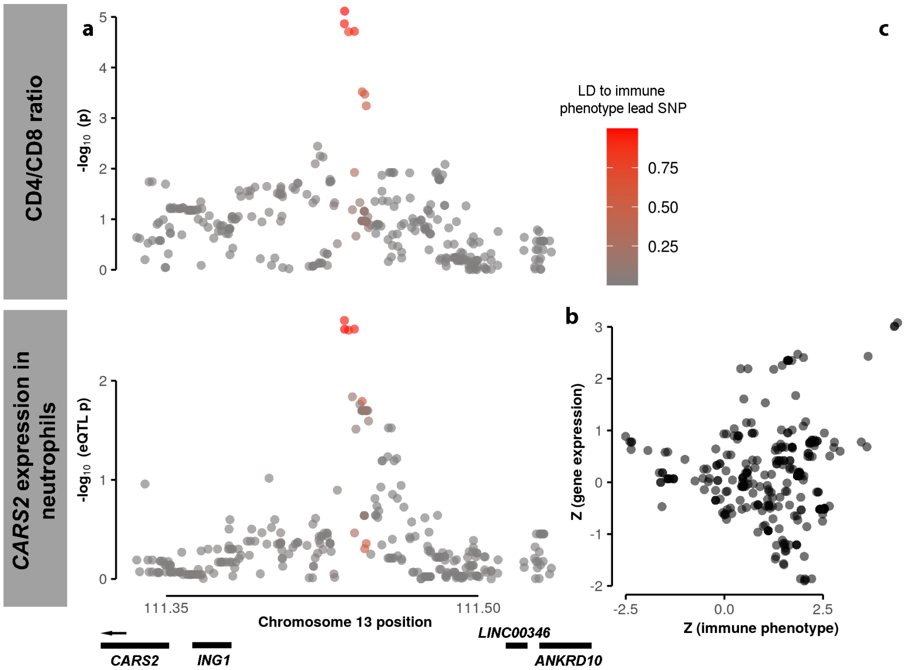

The association signal of CD4/CD8 ratio at a genetic locus on chromosome 13 is consistent with the association signal to *CARS2* expression in neutrophils (a, shaded by linkage disequilibrium (LD, r^2) to the immune phenotype lead single nucleotide polymorphism (SNP)). The association Z statistics of CD4/CD8 ratio and *CARS2* expression in neutrophils are strongly correlated (b). (c) shows the CD4/CD8 ratio and the level of *CARS2* expression in neutrophils per genotype of the lead variant rs9515281.

Supplementary Figure 10: Shared genetic association on chromosome 12 for *CLEC4C* expression in neutrophils and monocytes and the number of cDC3

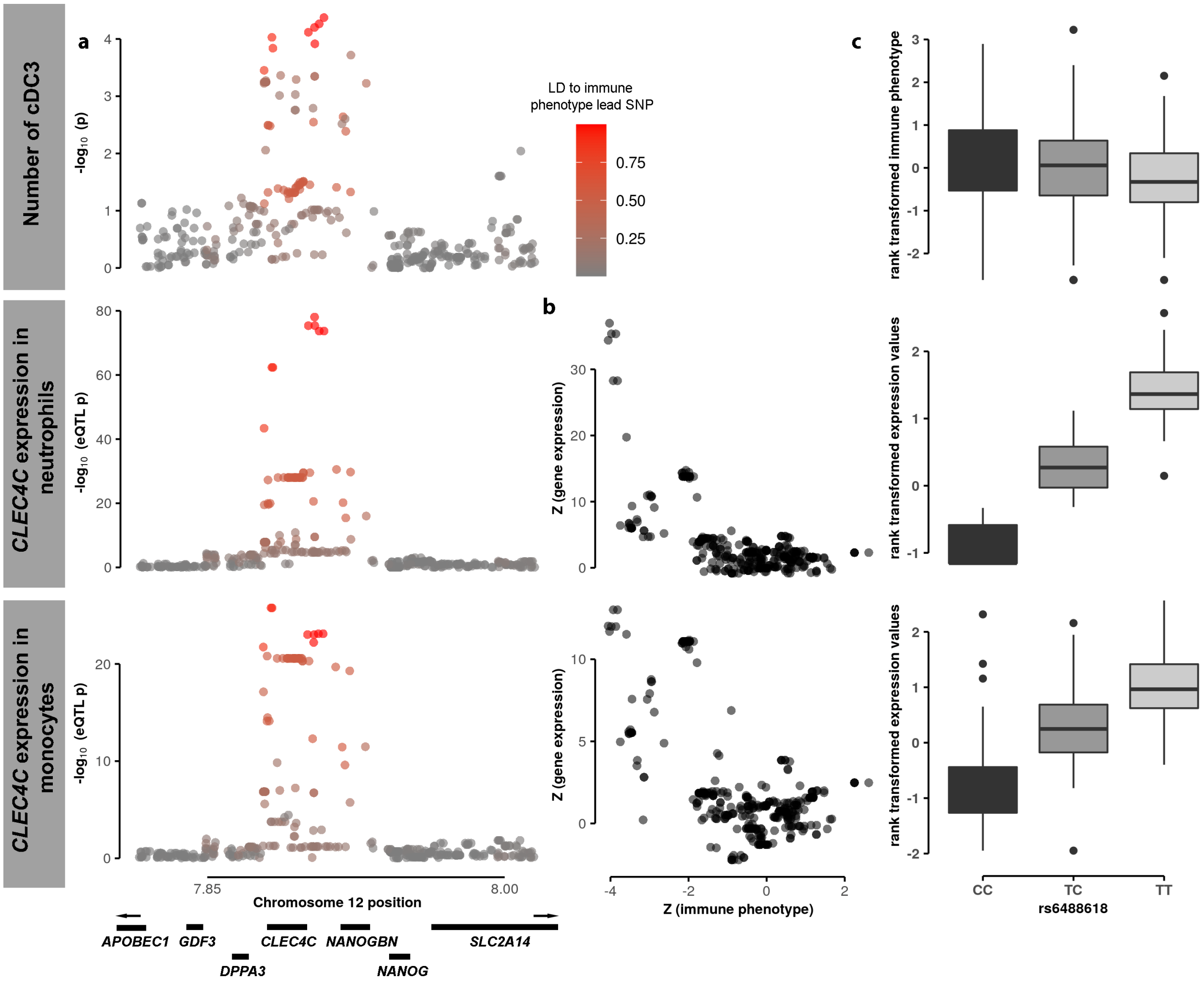

The association signal of the number of cDC3 cells at a genetic locus on chromosome 12 is consistent with the association signal to *CLEC4C* expression in neutrophils and monocytes (a, shaded by linkage disequilibrium (LD, r^2) to the immune phenotype lead single nucleotide polymorphism (SNP)). The association Z statistics of the number of cDC3 cells and *CLEC4C* expression in neutrophils and monocytes are strongly correlated (b). (c) shows the number of cDC3 cells and the level of *CLEC4C* expression in neutrophils and monocytes per genotype of the lead variant rs6488618.

Supplementary Figure 11: Shared genetic association on chromosome 12 for *ZNF780A* expression in T cells and monocytes and CD69 in HLA-DR+CD16+ NK cells

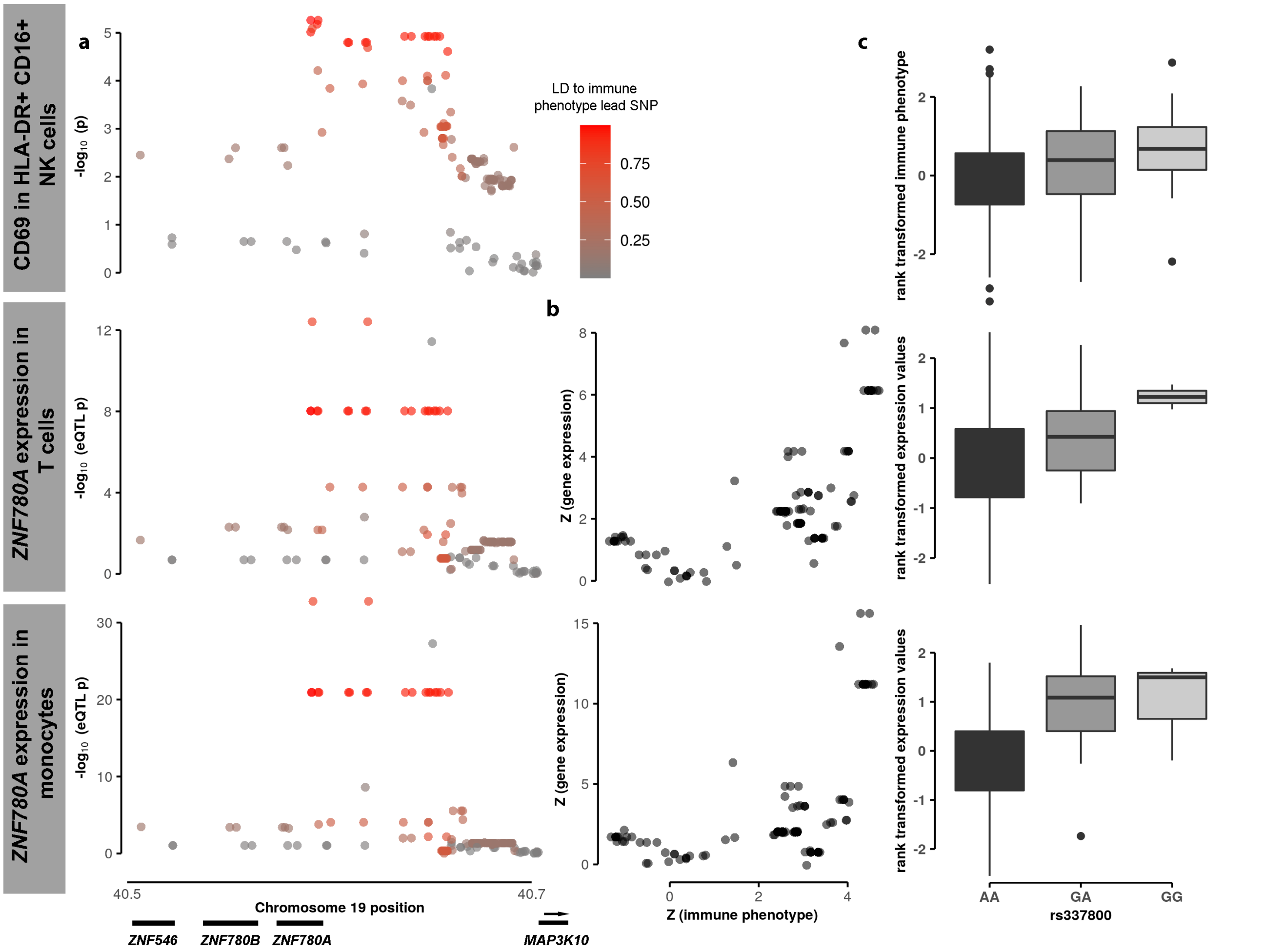

The association signal of mean fluorescence intensity (MFI) of CD69 in HLA-DR+ CD16+ natural killer (NK) cells at a genetic locus on chromosome 19 is consistent with the association signal to *ZNF780A* expression in T cells and monocytes (a). The association Z statistics of the MFI of CD69 in HLA-DR+ CD16+ NK cells and *ZNF780A* expression in T cells and monocytes are strongly correlated (b). (c) shows the MFI of CD69 in HLA-DR+ CD16+ NK cells and the level of *ZNF780A* expression in T cells and monocytes per genotype of the lead variant rs337800.

Supplementary Figure 12: Six immune phenotypes were not normally distributed after rank transformation

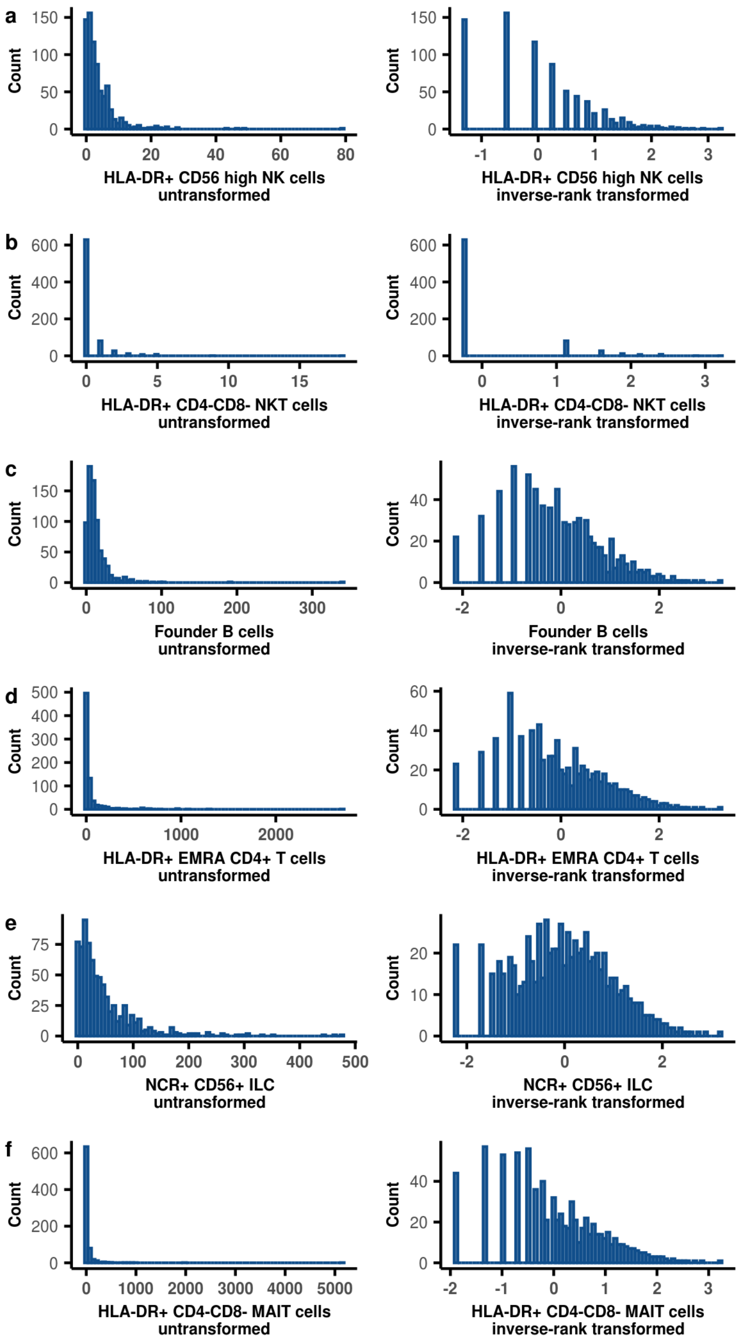

Six phenotypes were not normally distributed after rank transformation. After visual inspection, we removed two phenotypes - the number of HLA-DR+ CD56^{high} NK cells (a) and the number of HLA-DR+ CD4-CD8- NKT cells (b) from further analysis and decided to binarize two other phenotypes - the number of founder B cells (c) and the number of HLA-DR positive CD4 positive EMRA T cells (e). We did not perform any other normalization for the two remaining phenotypes - the number of NCR+ CD56+ innate lymphoid cells (e) and the number of HLA-DR+ CD4-CD8- MAIT cells (f).

Supplementary Figure 13: Converting two quantitative immune phenotypes to dichotomous traits

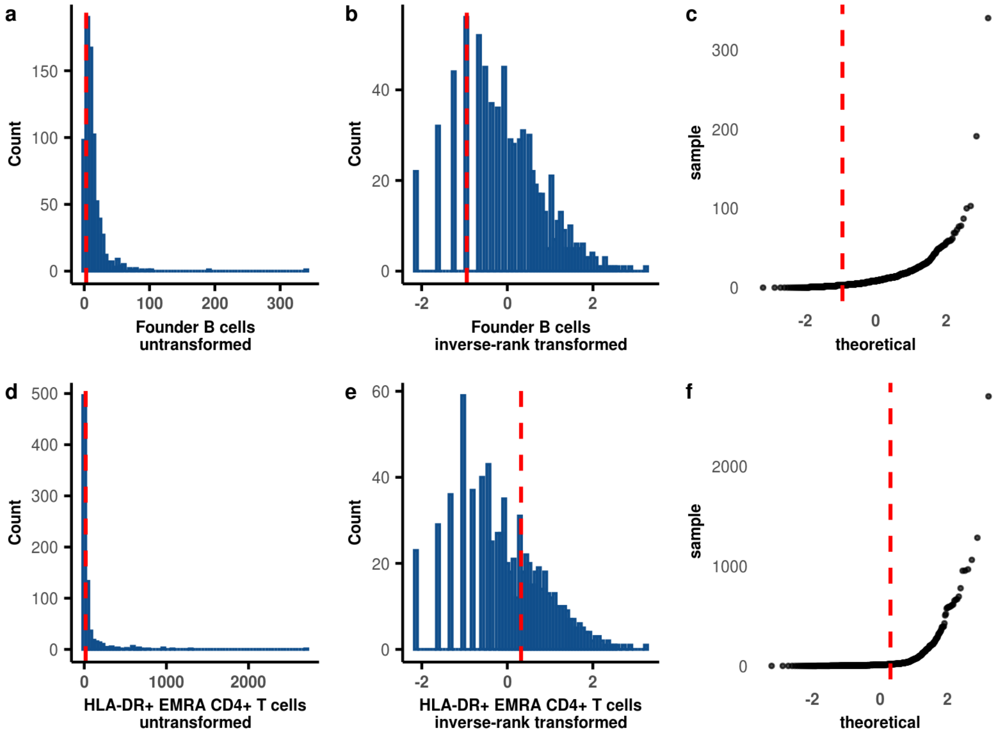

Two phenotypes - the number of founder B cells (panels a, b, and c) and the number of HLA-DR positive CD4 positive EMRA T cells (panels d, e, and f) - were binarized. Plots show the distribution of untransformed (a, d) as well as rank transformed (b, e) phenotypes and the quantile-quantile-plots (c, f). The thresholds for dichotomization (red dashed lines) were chosen as the point with the highest slope of the slope of the quantile-quantile plots.

Supplementary Figure 14: Schematic of the analysis windows used to identify shared genetic effects between immune phenotypes and gene expression traits

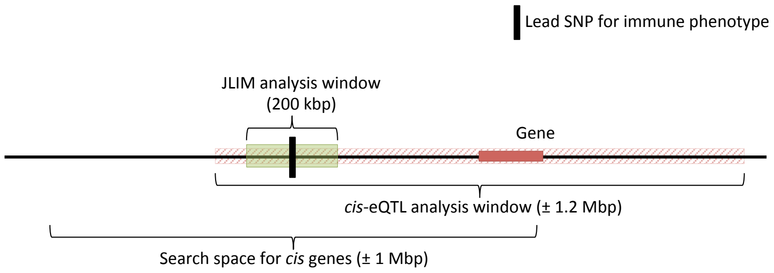

To identify effects shared between immune and gene expression phenotypes we first identified single nucleotide polymorphisms (SNPs) independently associated with an immune phenotype with association $p < 1\times10^{-5}$ (immune phenotype lead SNP). We then identified all genes with a transcription start site (TSS) within one megabase from that lead SNP. For each of these genes we performed a *cis*-expression quantiative trait locus (*cis*-eQTL) analysis to identify all indepently associated SNPs ($p < 1\times10^{-3}$) within 1.2 megabase from the TSS. For each gene with an eQTL with $p < 1\times10^{-3}$ within 200 kilobase from the immune phenotype lead SNP (JLIM analysis window) we then performed JLIM analysis on all SNPs within the JLIM analysis window to test for evidence of a shared genetic effect between the immune phenotype and gene expression traits.

Supplementary Table 1: Flow cytometry measurements for 166 immune phenotypes provided by the Milieu Intérieur project

Immunophenotype	Measure type
CD16hi NK cells	Absolute count
CD56hi NK cells	Absolute count
CD69+ CD16hi NK cells	Absolute count
CD69+ CD56hi NK cells	Absolute count
CD8a+ CD16hi NK cells	Absolute count
CD8a+ CD56hi NK cells	Absolute count
HLA-DR+ CD56hi NK cells	Absolute count
NK cells	Absolute count
CD16 in CD16hi NK cells	MFI
CD16 in CD56hi NK cells	MFI
CD69 in CD16hi NK cells	MFI
CD69 in CD56hi NK cells	MFI
CD69 in CD69+ CD16+ NK cells	MFI
CD69 in CD69+ CD56+ NK cells	MFI
CD69 in CD8a+ CD16+ NK cells	MFI
CD69 in CD8a+ CD56+ NK cells	MFI
CD69 in HLA-DR+ CD16+ NK cells	MFI
CD8a in CD16hi NK cells	MFI
CD8a in CD56hi NK cells	MFI
CD8a in CD69+ CD16+ NK cells	MFI
CD8a in CD69+ CD56+ NK cells	MFI
CD8a in CD8a+ CD16+ NK cells	MFI
CD8a in CD8a+ CD56+ NK cells	MFI
CD8a in HLA-DR+ CD16+ NK cells	MFI
HLA-DR in CD16hi NK cells	MFI
HLA-DR in CD56hi NK cells	MFI

HLA-DR in CD69+ CD16+ NK cells	MFI
HLA-DR in CD69+ CD56+ NK cells	MFI
HLA-DR in CD8a+ CD16+ NK cells	MFI
HLA-DR in CD8a+ CD56+ NK cells	MFI
HLA-DR in HLA-DR+ CD16+ NK cells	MFI
HLA-DR in HLA-DR+ CD56+ NK cells	MFI
NKp46 in NK cells	MFI
ratio of CD16 MFI in CD16hi to CD56hi NK cells	Ratio
ratio of CD16hi to CD56hi NK cells	Ratio
CD14hi monocytes	Absolute count
CD16hi monocytes	Absolute count
monocytes	Absolute count
CD16 in CD14hi monocytes	MFI
CD16 in CD16hi monocytes	MFI
basophils	Absolute count
eosinophils	Absolute count
neutrophils	Absolute count
CD16 in basophils	MFI
CD16 in eosinophils	MFI
CD16 in neutrophils	MFI
CD203c in basophils	MFI
CD32 in basophils	MFI
CD62L in eosinophils	MFI
CD62L in neutrophils	MFI
FcεRI in basophils	MFI
FcεRI in eosinophils	MFI
FcεRI in neutrophils	MFI
cDC1	Absolute count
cDC3	Absolute count
pDC	Absolute count

CD86 in CD14hi monocytes	MFI
CD86 in cDC1	MFI
CD86 in cDC3	MFI
CD86 in pDC	MFI
HLA-DR in CD14hi monocytes	MFI
HLA-DR in cDC1	MFI
HLA-DR in cDC3	MFI
HLA-DR in pDC	MFI
CD161+ ILC3	Absolute count
CD56+ ILC	Absolute count
ILC	Absolute count
ILC1	Absolute count
ILC2	Absolute count
ILC3	Absolute count
NCR- CD56+ ILC	Absolute count
NCR+ CD56+ ILC	Absolute count
CD127 in ILC1	MFI
CD161 in ILC1	MFI
CD161 in ILC3	MFI
CD4+ T cells	Absolute count
CD8b- CD4- T cells	Absolute count
CD8b+ T cells	Absolute count
CM CD4+ T cells	Absolute count
CM CD8+ T cells	Absolute count
EM CD4+ T cells	Absolute count
EM CD8+ T cells	Absolute count
EMRA CD4+ T cells	Absolute count
EMRA CD8+ T cells	Absolute count
HLA-DR+ CM CD4+ T cells	Absolute count
HLA-DR+ CM CD8+ T cells	Absolute count

HLA-DR+ EM CD8+ T cells	Absolute count
HLA-DR+ EMRA CD4+ T cells	Absolute count
HLA-DR+ EMRA CD8+ T cells	Absolute count
HLA-DR+ in EM CD4+ T cells	Absolute count
naïve CD4+ T cells	Absolute count
naïve CD8+ T cells	Absolute count
T cells	Absolute count
CCR7 in CM CD4+ T cells	MFI
CCR7 in CM CD8+ T cells	MFI
CCR7 in EM CD4+ T cells	MFI
CCR7 in EM CD8+ T cells	MFI
CCR7 in EMRA CD4+ T cells	MFI
CCR7 in EMRA CD8+ T cells	MFI
CCR7 in naïve CD4+ T cells	MFI
CCR7 in naïve CD8+ T cells	MFI
CD4:CD8 ratio	Ratio
activated Treg	Absolute count
conventional T cells	Absolute count
memory Treg	Absolute count
naïve Treg	Absolute count
Treg cells	Absolute count
ICOS in activated Treg	MFI
ICOS in memory Treg	MFI
ICOS in naïve Treg	MFI
CD4- CD8- MAIT cells	Absolute count
CD4- CD8- NKT cells	Absolute count
CD8+ MAIT cells	Absolute count
HLA-DR+ CD4- CD8- MAIT cells	Absolute count
HLA-DR+ CD4- CD8- NKT cells	Absolute count
MAIT cells	Absolute count

NKT in T cells	Absolute count
γδ+ T cells	Absolute count
B cells	Absolute count
CD21- CD27- B cells	Absolute count
CD21- CD27+ B cells	Absolute count
CD24hi memory B cells	Absolute count
CD24low memory B cells	Absolute count
double negative memory B cells	Absolute count
founder B cells	Absolute count
germinal center B cells	Absolute count
IgM- marginal zone B cells	Absolute count
IgM+ marginal zone B cells	Absolute count
marginal zone B cells	Absolute count
memory B cells	Absolute count
naïve B cells	Absolute count
transitional B cells	Absolute count
CD19 in B cells	MFI
CD21 in B cells	MFI
CD21 in CD24hi memory B cells	MFI
CD21 in CD24int memory B cells	MFI
CD21 in CD24low memory B cells	MFI
CD21 in double negative memory B cells	MFI
CD21 in germinal center B cells	MFI
CD21 in IgM- marginal zone B cells	MFI
CD21 in IgM+ marginal zone B cells	MFI
CD21 in marginal zone B cells	MFI
CD21 in memory B cells	MFI
CD21 in naïve B cells	MFI
CD21 in transitional B cells	MFI
CD24 in B cells	MFI

CD24 in double negative memory B cells	MFI
CD24 in germinal center B cells	MFI
CD24 in IgM- marginal zone B cells	MFI
CD24 in IgM+ marginal zone B cells	MFI
CD24 in marginal zone B cells	MFI
CD24 in memory B cells	MFI
CD24 in naïve B Cells	MFI
CD27 in B cells	MFI
CD38 in B cells	MFI
CD38 in double negative memory B cells	MFI
CD38 in germinal center B cells	MFI
CD38 in memory B cells	MFI
IgD in B cells	MFI
IgM in B cells	MFI
CCR6+ CD4+ T cells	Absolute count
CCR6+ CD8+ T cells	Absolute count
CRTh2+ CD4+ T cells	Absolute count
CXCR5+ CD4+ T cells	Absolute count
CD45+ cells	Absolute count
total number of cells	Absolute count

Supplementary Table 2: Polygenic risk score (PRS) results without conditioning on shared genetic effects

eQTL significance threshold (*p*-value)	Number of variants in gene expression trait PRS (mean, SD)	Number of trait pairs with shared genetic effect / Number of colocalized trait pairs	Number of trait pairs with significant FDR-adjusted PRS p-value (with shared effect / colocalized)	Mean trait variance explained by gene expression PRS in trait pairs with shared vs. colocalized associations	Difference in trait variance explained by gene expression PRS in shared vs. colocalized associations (Mann-Whitney test, *p*-value)	Evidence of shared association predicting immune trait variance explained by gene expression PRS (univariate regression R^2 [confidence interval], *p*-value)
5×10^{-8}	1.42, 0.75	118/8,134	68 (57.6%) / 282 (3.5%)	2.52×10^{-02} / 2.43×10^{-03}	**3.56×10^{-35}**	**0.160 [0.146-0.175], < 1×10^{-100}**
1×10^{-7}	1.47, 0.81	125/8,781	72 (57.6%) / 285 (3.3%)	2.55×10^{-02} / 2.43×10^{-03}	**1.62×10^{-36}**	**0.157 [0.143-0.171], < 1×10^{-100}**
5×10^{-7}	1.79, 1.05	155/11,380	67 (43.2%) / 240 (2.1%)	1.95×10^{-02} / 2.19×10^{-03}	**2.52×10^{-30}**	**0.120 [0.109-0.131], < 1×10^{-100}**
1×10^{-6}	2.11, 1.26	168/13,237	64 (38.1%) / 191 (1.4%)	1.76×10^{-02} / 2.02×10^{-03}	**7.46×10^{-32}**	**0.120 [0.110-0.130], < 1×10^{-100}**
5×10^{-6}	5.06, 2.36	190/16,306	33 (17.4%) / 59 (0.4%)	1.06×10^{-02} / 1.68×10^{-03}	**7.23×10^{-24}**	**0.070 [0.062-0.077], < 1×10^{-100}**
1×10^{-5}	8.87, 3.15	190/16,460	21 (11.1%) / 22 (0.1%)	7.82×10^{-03} / 1.57×10^{-03}	**3.70×10^{-24}**	**0.053 [0.046-0.060], < 1×10^{-100}**
5×10^{-5}	36.84, 6.45	190/16,462	2 (1.1%) / 0 (0.0%)	3.73×10^{-03} / 1.48×10^{-03}	**2.48×10^{-08}**	**0.009 [0.006-0.012], 2.48×10^{-35}**
1×10^{-4}	69.47, 8.86	190/16,462	1 (0.5%) / 0 (0.0%)	2.82×10^{-03} / 1.43×10^{-03}	**6.44×10^{-06}**	**0.004 [0.002-0.006], 2.04×10^{-16}**
1×10^{-3}	570.24, 26.17	190/16,462	0 (0.0%) / 1 (0.0%)	1.72×10^{-03} / 1.34×10^{-03}	5.26×10^{-02}	0.000 [0.000-0.000], 1.36×10^{-01}
1×10^{-2}	4292.85, 73.14	190/16,462	0 (0.0%) / 0 (0.0%)	1.41×10^{-03} / 1.33×10^{-03}	8.38×10^{-02}	0.000 [0.000-0.000], 3.22×10^{-01}

We considered 22,379 gene expression/immune trait pairs in our analysis. For each, we identified all independent variants meeting a threshold of association in the eQTL trait. We used these variants to calculate polygenic risk scores (PRS) for each individual in the Milieu Intérieur project immune phenotype collection, and calculated the proportion of paired immune trait variance explained by that PRS (R^2_{PRS}). We compared R^2_{PRS} values for trait pairs with and without evidence for a shared underlying genetic effect using a Mann-Whithney-Wilcoxon test and determined the associations of JLIM *p*-values with R^2_{PRS} values and PRS *p*-values using univariate linear regression. Statistically significant results are shown in bold font.
