# Supplementary material for: Shared associations identify causal relationships between gene expression and immune cell phenotypes
Source: Commun Biol. 2021 Mar 4;4:279. doi: 10.1038/s42003-021-01823-w (PMC7933159; doi:10.1038/s42003-021-01823-w)
Supplement: Supplementary file 3 — Description of Additional Supplementary Files [file 42003_2021_1823_MOESM3_ESM.pdf]

Description of Additional Supplementary Files

File name: Supplementary Data 1-4

Description: The source data underlying figures 1-4 can be found in the supplementary excel files named Supplementary Data 1-4, respectively. The data for each figure panel is shown in a separate sheet.

File name: Supplementary Data 5

Description: Excel copy of Supplementary Table 1
